# HIV vaccines in 2022: where to from here?

**DOI:** 10.1002/jia2.25923

**Published:** 2022-05-17

**Authors:** Stacey Hannah, Kundai Chinyenze, Robin Shattock, Ntando Yola, Mitchell Warren

**Affiliations:** ^1^ AVAC New York New York USA; ^2^ IAVI Nairobi Kenya; ^3^ Imperial College London London UK; ^4^ Desmond Tutu Health Foundation and Advocacy for Prevention of HIV and AIDS (APHA) Cape Town South Africa

1

For over 2 years, the world has been riveted by progress and pitfalls of the COVID‐19 pandemic and subsequent vaccine development and rollout. However, alongside scientific milestones of COVID‐19 vaccines, there have been significant results from HIV vaccine and antibody‐mediated prevention trials. These include the first proof of concept of HIV prevention from passive antibodies [1] and recent insights on the possibility of germline targeting and the mRNA platform towards an effective HIV vaccine [2].

But HIV vaccine research has also had set‐backs, with key trials stopped for non‐efficacy [3, 4]. While there is hope that lessons from COVID‐19 vaccines can be applied to HIV vaccine development, the field confronts scientific challenges that may be more daunting than ever.

Given the backdrop of COVID vaccine development and the broader HIV prevention landscape, how should HIV vaccine research advance? The way forward requires all stakeholders—funders, researchers, product developers, trial networks and advocates—to analyse closely what the field has learned to date, develop clarity on the critical scientific challenges and agree on a coordinated strategy to pursue answers.

Since early 2020, two trials of different HIV vaccine candidates and two trials testing antibody‐mediated prevention (with implications for vaccines) showed no overall efficacy in preventing HIV acquisition. However, all four trials yielded important information. The AMP trials confirmed that a broadly neutralizing antibody (bnAb) can offer efficacy, when the circulating variants of HIV are highly sensitive to the bnAb neutralization. Because only a minority of circulating strains associated with incident HIV infection were neutralized by the single VRC01 bnAb, these studies suggest that a combination of bnAbs is needed to achieve broad protection. As multiple research groups pursue different antibody combinations, the field must collaboratively develop and articulate criteria for selecting combination bnAb products for future efficacy trials.

Similarly, results showing no efficacy from both the Uhambo and Imbokodo trials re‐focus the questions that must be posed next in HIV vaccine development. While Imbokodo focused on cisgender women, a sibling trial, MOSAICO, which recruited gay men and other men who have sex with men and MSM and transgender women, continues, in hopes that differences in vaccine inserts and mode of exposure might result in some demonstration of efficacy.

For the first time in years, there is no HIV vaccine efficacy trial with a clear product development plan on the horizon. While this pipeline may seem daunting, now is the opportune moment to consolidate what has been learned from recent results, accelerate investigation of new approaches and chart a novel path forward for both science and collaboration across stakeholders.

More recently, a series of smaller trials have generated excitement around germline targeting, a strategy using sequential vaccines to prompt B cells to mature into bnAbs. Various research teams are also exploring a range of new antigens to elicit bnAbs using the mRNA platform that proved successful for COVID‐19 vaccines.

Noting the excitement around these approaches, it is important to manage expectations: to date, only small phase 1 studies have been conducted. While the mRNA platform proved successful for COVID‐19, how to elicit a protective immune response against HIV remains unknown and poses one of the most significant challenges in biomedical science. mRNA technology may be an important step forward to speed identification of the right target antigens for a protective response, but it does not address inherent immunological challenges associated with HIV envelope. It is important, therefore, to ensure additional approaches, alternative immunogen designs and T‐cell immunity are also pursued.

Critical questions the field must confront include: Is it possible to develop effective, long‐lasting bnAbs? What concentration of bnAbs are needed for a meaningful duration of protection? Can bnAbs alone prevent HIV acquisition, or will a complementary role for T cells be required? What results will an mRNA platform deliver? What will it take to test an array of strategies simultaneously to pursue answers and accelerate timelines?

Given the scientific challenges, the urgency for an HIV vaccine and the high HIV incidence consistently seen across efficacy trials, it is important the field does not shift from one concept to another and, instead, accelerate testing of multiple approaches in innovative and iterative human clinical trials, especially given flat HIV vaccine funding [5]. A number of research groups and funders are turning to experimental medicine vaccine trials. Designed as small phase 1 trials, but setting aside the necessity to aim for a licensable product, these trials aim to quickly and safely answer if an experimental agent induces a potentially protective response, and may offer researchers a more viable way to shift through the many variables that may contribute to successful HIV vaccine design.

The pursuit of these vaccine approaches must also take into account historic advances in antiretroviral‐based protection. Recent regulatory approvals of the dapivirine vaginal ring and injectable cabotegravir for pre‐exposure prophylaxis (PrEP) offer additional options for HIV prevention. This expanded “method mix” presents tremendous opportunities to reduce global incidence of HIV. Advocates are demanding these products are integrated into policies and programs, which must be well‐funded and community‐led. These efforts are fundamental, or proven interventions will lay idle—and lessons from COVID‐19 make this plain. COVID‐19 vaccine development leveraged unprecedented global coordination and scientific knowledge from decades of research on HIV, cancer and other coronaviruses. The result was a suite of effective vaccines developed in months, instead of years or decades, but the lack of a global commitment to equitable access has inhibited impact.

As PrEP options become available, it will also affect how to define the ideal characteristics of a vaccine or antibody combination. A vaccine's target product profile will not only be considered against other vaccine candidates but may need to out‐perform 2‐ or 6‐month PrEP injections. The expanding PrEP landscape will also require ever more innovation in trial design, keeping in mind the unknown timeline to the field's next efficacy trial. The phase IIb PrEPVacc trial provides a window on one version of this future, testing two vaccine strategies in tandem with two formulations of oral PrEP [6]. Especially in high HIV incident settings, the frequency of HIV exposure might overwhelm the immune response mounted by a vaccine alone, and the chemical shield of PrEP could shore up protection. With new PrEP options could come new opportunities to build potential PrEP–vaccine combinations, a subject for the entire HIV prevention field to consider.

While almost four decades of research have yet to deliver a licensed HIV vaccine, they have been an engine of discovery, providing vaccine know‐how, technology, clinical trial network and site infrastructure, researchers and advocates that galvanized the development of multiple COVID‐19 vaccines in record time. SARS‐CoV‐2 proved to be a far easier vaccine target than HIV, but even so, the response to COVID‐19 has shown that timelines can be compressed and new technologies can be developed, tested and distributed quickly—at least for wealthier nations. The field must face the challenges ahead with honest reflection, innovation, speed and clarity. The field must confront what it has learned—and not learned—from the science to‐date, and generate new hypotheses, fresh ideas and novel strategies to what is tested, and how. And when an HIV vaccine is finally licensed, the most important work begins—delivering it with equity, confidence and trust.

## COMPETING INTERESTS

The authors declare that they have no competing interests.

## AUTHORS’ CONTRIBUTIONS

MW and SH drafted the initial version of the manuscript. All authors reviewed and revised the manuscript. All authors approved the final version of the manuscript.

## FUNDING

MW and SH were supported by the Coalition to Accelerate and Support Prevention Research (CASPR), made possible by the generous support of the American people through the US President's Emergency Plan for AIDS Relief (PEPFAR) and the US Agency for International Development (USAID).

## DISCLAIMER

The views expressed are those of the authors, and the contents do not necessarily reflect the views of PEPFAR, USAID or the United States Government.

## References

[1] Corey L , Gilbert PB , Juraska M , Montefiori DC , Morris L , Karuna ST , et al. Two randomized trials of neutralizing antibodies to prevent HIV‐1 acquisition. N Engl J Med. 2021;384:1003–14. 33730454 10.1056/NEJMoa2031738 PMC8189692

[2] Kim J , Vasan S , Kim JH , Ake JA . Current approaches to HIV vaccine development: a narrative review. J Int AIDS Soc. 2021;24(S7):22–34. 10.1002/jia2.25793 PMC8606871 34806296

[3] Gray GE , Bekker LG , Laher F , Malahleha M , Allen M , Zoe M , et al. Vaccine efficacy of ALVAC‐HIV and bivalent subtype C gp120‐MF59 in adults. N Engl J Med. 2021;384:1089–100. 33761206 10.1056/NEJMoa2031499 PMC7888373

[4] National Institutes of Health Press Release . HIV vaccine candidate does not sufficiently protect women against HIV infection. 31 August 2021.

[5] ResourceTracking for HIV Prevention R&D Working Group . 2020. HIV prevention research & development investments. https://www.hivresourcetracking.org/investment‐by‐technology/. Accessed 20 April 2022.

[6] Joseph S , Kaleebu P , Ruzagira E , Hansen C , Seeley J , Basajja V , et al. OC 8491 PrEPVacc: a phase III, MAMS adaptive prophylactic HIV vaccine trial with a second randomisation to compare F/TAF with TDF/FTC PrEP. BMJ Glob Health. 2019;4:A10.

